# Is motor asymmetry a predictor of non-motor outcomes in Parkinson’s disease?

**DOI:** 10.1007/s00702-025-03080-1

**Published:** 2026-01-12

**Authors:** Philippe Voruz, Marc Vérin, Julie Anne Péron

**Affiliations:** 1 Clinical and Experimental Neuropsychology Laboratory, Faculty of Psychology, Geneva, Switzerland; 2 Neurosurgery Department, Geneva University Hospitals and Faculty of Medicine, Geneva, Switzerland; 3 Geospatial Molecular Epidemiology Group, Laboratory for Biological Geochemistry, School of Architecture, Civil and Environmental Engineering, Ecole Polytechnique Fédérale de Lausanne, Lausanne, Switzerland; 4 Neurology Department, Orléans University Hospital, Orléans, France; 5 Brain-Clinical and Experimental Neuroplasticity, LI2RSO, University of Orléans, Orléans, France; 6 Neurology Department, Geneva University Hospitals and Faculty of Medicine, Geneva, Switzerland; 7 Faculty of Psychology and Educational Sciences, 40 bd du Pont d’Arve, Geneva, 1205 Switzerland

**Keywords:** Motor symptom asymmetry, Non-motor Symptoms, Cognition, Psychiatry; Psychiatry, Emotion

## Abstract

The objective of this opinion paper is to highlight, based on recent studies, the importance of considering motor symptom (a)symmetry in the progression and management of non-motor symptoms in Parkinson’s disease.

Parkinson’s Disease (PD) has long been characterized by its cardinal motor symptoms: bradykinesia, tremor, rigidity, and postural instability, typically presenting with asymmetrical onset (Djaldetti et al. 2006). However, this motor asymmetry is more than a diagnostic hallmark; it may be a window into the neurocognitive and neuropsychiatric future of the patient (for review see, Voruz et al. 2025b). While PD has traditionally been approached as a motor disorder, accumulating evidence calls for a paradigm shift: motor symptom (a)symmetry should be recognized as a key predictor of non-motor outcomes, particularly cognitive decline, psychiatric burden, and emotion recognition deficits. Within this framework, right-predominant PD (RPD) patients tend to show more pronounced cognitive decline, particularly in executive functioning and memory domains (Harris et al. 2013, 2015; Voruz et al. 2022, 2023; Fiorenzato et al. 2021). In contrast, left-predominant PD (LPD) has been linked to affective and socio-emotional symptoms such as anxiety, depression, and reduced emotion recognition (Béreau et al. 2023; Foster et al. 2010, 2011; Benis et al. 2020; Voruz et al. 2020, 2025a; Stirnimann et al. 2018).

Motor symptom asymmetry thus emerges as a potential predictor not only of cognitive outcomes but also of affective and socio-emotional functioning. It is important to distinguish between side of onset and motor symptom asymmetry. The side of onset refers to the body side first affected by motor symptoms, whereas motor asymmetry reflects the current degree of difference between the two sides, which may change with disease progression and treatment (Djaldetti et al. 2006; Voruz and Péron 2025). Although these features are related, they capture distinct aspects of disease expression, and current evidence does not suggest that one side of onset inherently corresponds to greater or lesser degrees of asymmetry. Motor symptom asymmetry thus emerges as a potential predictor not only of cognitive outcomes but also of affective and socio-emotional functioning. Moreover, recent models of PD onset and evolution, such as the “α-Synuclein Origin and Connectome Model” (SOC Model; Borghammer 2021) have emphasized the clinical and biological significance of asymmetrical motor presentations. Neuroimaging, clinical and neuropathological studies have demonstrated that the hemisphere contralateral to the more affected side often shows greater dopaminergic depletion and cortical thinning (Cheesman et al. 2005); Danti et al. 2015; Santos et al. 2016), supporting the association, for instance, between RPD and relatively greater left-hemispheric neurodegeneration. However, while this pattern is frequently observed, it is not universal, and individual variability in hemispheric pathology remains considerable (Ocklenburg et al. 2024; Lubben et al. 2021).

Yet, despite its clinical relevance, symmetry remains under-analyzed in PD research, largely due to the absence of standardized thresholds for determining motor asymmetry. Many studies fail to classify or analyze symmetric profiles systematically, resulting in a significant blind spot, especially considering that patients with symmetric motor onset may represent a more globally degenerated, “brain-first” subtype with more pronounced cognitive decline.

This limitation has been recently addressed by our group, who emphasized that while motor asymmetry is a key feature of PD, there has been no consensus on the best method or threshold to define asymmetry, thereby hampering consistent stratification in clinical and research settings (Voruz et al. 2025b; Voruz and Péron 2025). Their work used data from the Parkinson’s Progression Markers Initiative (PPMI) to evaluate the predictive validity of both standardized and non-standardized asymmetry scores against dopaminergic damage in the putamen, measured via DaTSCAN. Both scores were significant predictors, with the non-standardized score achieving an AUC of 0.621 (*p* < .001) and an optimal cut-off of ± 2.50 (sensitivity 0.874; specificity 0.783). The standardized score yielded an AUC of 0.569 (*p* = .018), with a cut-off of ± 0.188 (sensitivity 0.908; specificity 0.823) (Voruz and Péron 2025). These findings represent an important advance toward refining diagnostic and research thresholds, facilitating more accurate categorization of symmetric and asymmetric motor presentations.

Failure to account for these profiles may obscure important clinical differences: patients who are currently symmetric in their motor presentation often exhibit faster cognitive decline and broader neurodegeneration, while asymmetric patients show hemisphere-specific cognitive or psychiatric outcomes (Ocklenburg et al. 2024; Voruz et al. 2025b; Nuber-Champier et al. 2024; Borghammer 2021; Borghammer et al. 2021, 2022; Borghammer and Van Den Berge 2019; Fedorova et al. 2023; Hansen et al. 2025). It is crucial to note, however, that motor symmetry at a given time point is not equivalent to bilateral onset of symptoms. Some individuals who were initially asymmetric may evolve toward symmetry as the disease progresses, whereas others may present bilaterally from the outset. These trajectories likely reflect distinct pathophysiological mechanisms that should be considered separately in future research (Voruz and Péron 2025).

We advocate for the systematic inclusion of asymmetry profiles in clinical evaluation and follow-up. Neuropsychological monitoring should be tailored: RPD patients require early cognitive intervention, while LPD patients benefit from psychiatric support, particularly addressing emotion recognition deficits, mood symptoms, and social cognition impairments. Symmetric cases warrant close cognitive surveillance. To operationalize this approach, we recommend the initiation of an international consensus, and the development of a clinically integrated decision tree. Patients would be classified by dominant motor symptom presentation (RPD, LPD, or symmetric), then stratified using neuroimaging biomarkers (e.g., cortical thinning, white matter asymmetries, dopamine depletion), and complemented by domain-specific neuropsychological assessment. Such a framework would enable targeted, personalized interventions, like cognitive rehabilitation, psychiatric care, and individualized therapy, enhancing quality of life and potentially delaying neurodegenerative progression. In addition, factors such as gender (Constantin et al. 2023), as well as types of medication and therapeutic interventions (Voruz et al. 2025a), should be systematically integrated and examined within this framework. A greater granularity of clinical profiles among individuals living with PD is not only possible but likely, and should be anticipated in future research. Moreover, future research should continue to disentangle side of onset and degree of asymmetry, as these may reflect partially independent biological processes with distinct prognostic implications.

In conclusion, motor symptom asymmetry is not a superficial feature, it is a powerful, multidimensional clinical marker. It predicts the trajectory of both cognitive and psychiatric symptoms, including emotion recognition impairments, and should be fully integrated into risk stratification and therapeutic planning. The recent establishment of thresholds for motor asymmetry is a critical step toward enabling more rigorous, evidence-based use of this marker in both research and clinical practice.
